# Recipients’ and providers’ perspectives of obesity and potential barriers to weight management programmes in patients with Rheumatoid Arthritis (RA): a qualitative study

**DOI:** 10.1186/s40608-017-0169-x

**Published:** 2017-10-18

**Authors:** G. Colligan, J. Galloway, H. Lempp

**Affiliations:** 1 Centre for Primary Care and Public Health, Blizard Institute, Barts and The London School of Medicine and Dentistry, Queen Mary University of London, London, UK; 2 Department of Academic Rheumatology, Kings College London, Weston Education Centre, 10 Cutcombe Road, London, SE5 9RJ UK

**Keywords:** Experiences, Obesity, Perspectives, Qualitative research method, Rheumatoid arthritis, Weight management

## Abstract

**Background:**

The UK rheumatology community serves an ageing and ethnically diverse population, with a growing public health concern about obesity. Overweight and obesity contribute to 2.8 million preventable deaths annually. A raised Body Mass Index (BMI) in those with Rheumatoid Arthritis (RA) can have a significant negative impact on clinical outcomes. The aim of the study was to examine patients’ and providers’ perceptions of obesity and potential barriers to participation in a future weight management programme to contribute to an appropriate intervention design.

**Method:**

Qualitative semi-structured interviews were carried out with 11 patients with RA and one focus group was held with 8 members of a multi-disciplinary team working in one Rheumatology outpatient clinic. Framework analysis (FA) contributed to the inductive thematic analysis, and was employed to assist with the identification of the emergent codes and final themes.

**Results:**

Three core themes were ascertained from the semi-structured interviews: i) The psychosocial impact of living with RA and obesity, ii) Challenges of living with RA and obesity and iii) Considerations for future weight management programmes. The Focus group analysis also identified three core themes: i) Micro-dynamics between patient and provider, ii) The relationship between the provider and the host institution in relation to the development of a future weight management programme and iii) The social and political context of obesity as a public health concern.

**Conclusion:**

Perceptions of obesity and weight gain and associated barriers to participating in weight management programmes, differ significantly between patients and providers. Patients, require a holistic approach to weight management by clinicians and the acknowledgement of the significant psychosocial impact of a dual diagnosis of RA and being overweight or obese. In contrast, providers seem reluctant to address weight increase with patients and require education and support at an individual and institutional level to integrate weight management into routine care.

## Background

Across the UK, obesity prevalence is currently 26.9% for both men and women [1]. In South London obesity is slightly below the national average of 24.9%; at 21% however, there is a high incidence of physical conditions where obesity is associated as a risk factor for the development of diabetes and cardiovascular disease, for example [2].

In RA, the burden of obesity may be greater still, as the inflammatory nature of the disease results in an apparent sarcopenia, with a reduction in muscle mass and relative increase in fat mass [3] resulting in underestimation of obesity when measures such as the BMI are employed [4]. Work by Stavropoulos (2007) suggests that a BMI >28 kg/m^2^ in patients with RA could constitute obesity, thus adding more RA to the obese category and improving the predictive value of this indicator in RA [3, 5].

Overweight and obese patients demonstrate poorer response to anti-TNF therapies and have worse clinical outcomes in terms of function and co-morbidities [4, 6]. They experience significantly worse pain and a higher frequency of past and current use of biologic drugs [7]. A higher prevalence of chronic pain and increased depressive symptoms were reported in overweight and obese people with RA [8], highlighting the potential difficulty for patients to engage in health promoting behaviour [9].

Work by Nikiphorou et al. (2016) on 2 large UK based RA inception cohorts, examined trends at first presentation of RA, including co-morbidities, and showed a significant increase in body mass index (0.15 units/year; 95% CI 0.11, 0.18), resulting in an increase in the prevalence of obesity at diagnosis from 13.3% in 1990 to 33.6% in 2010 [10].

Analysis of our local RA population as part of a quality improvement project supports these findings, where the time trend for the overall RA population revealed that weight was increasing by approximately 0.2 kg/year, with 64% of patients above normal weight, 31% overweight, 27% obese and 6% morbidly obese. The most notable change was in those in the overweight category (BMI >25-30 kg/m^2^), in whom average weight was increasing by 0.6 kg/year. Higher BMI was associated with higher disability and higher disease activity. The odds ratio for being in a state of low disease activity in obese patients compared to non-obese was 0.39 (95% CI 0.27, 0.56) [unpublished communication James Galloway].

Two unpublished patient surveys carried out in the local Rheumatology clinic, to explore attitudes to food and views about physical and emotional barriers to weight loss with RA, revealed that the majority of respondents (49% vs. 80%), indicated that patients had not taken part in any weight management programme since being diagnosed with RA and only 18% of patients engage in regular significant physical activity 3 or more times a week. More than one third of respondents described themselves as emotional eaters, who reach for food to feel better when emotional stress becomes too much [11].

Research addressing preventative aspects of weight loss on the musculoskeletal system is therefore urgent [12]. An unanswered question is whether treatment of obesity in the context of RA has particular requirements, or can we adopt the same approach for addressing obesity as in the wider population?

Qualitative research is a structured exploratory approach to data gathering, attempting to understand and explain particular phenomenon from the viewpoint of the individual, thus gaining deeper insight into the individual experience [13, 14].Bringing patients and providers together to understand more about what matters to them can lead to the identification of shared beliefs [15] so that healthcare services address issues important to service users [2].

Against this background of increasing weight gain within our local RA population, we therefore decided to carry out a qualitative study on patients (BMI >25 kg/m^2^) living with RA, and their clinicians, with the aim of systematically capturing first hand perspectives of the management of obesity and the potential barriers to weight management interventions.

## Methods

The study was carried out with patients and staff in one Rheumatology outpatient’s clinic in London, UK, which serves an ethnically diverse inner-city population.

### Participant selection and recruitment

Over a period of 3 months the direct care team provided a list of eligible patients (convenient sample) [16] for the researcher (GC) with the following inclusion criteria: i) adult with a diagnosis of RA > 2 years, ii) BMI > 25 kg/m^2^ (those in both the overweight and obese category) and iii) the ability to communicate in English. Potential participants were also identified, by GC, during weekly rheumatology outpatient clinics. An Invitation letter and Patient Information Sheet was given to each attendee, who expressed an interest to take part in the interview study. Permission was sought to contact the patient after forty-eight hours to arrange a mutually convenient time for a face to face interview.

Recruitment for the focus group was initiated by a Rheumatology consultant (JG), who invited eligible staff members to take part, with the following inclusion criteria: professional qualification, working within a Rheumatology speciality for >3 months.

### Data collection and analysis

Prior to recruitment a draft interview schedule based on findings from the literature [12, 17] and related work within the department, was piloted with one patient, who confirmed that questions were relevant, comprehensive and the length of the interview appropriate. To facilitate comparison with providers, a similar schedule was drawn up for the focus group. The questions broadly addressed, i) what participants understood by the term obesity, ii) what they perceived as potential barriers to weight management programmes and iii) what, in their experience might help to maintain weight loss long-term.

Semi-structured interviews have the advantage of examining predefined topics, whilst retaining flexibility, for participant and researcher to explore issues in depth [18]. Due to the limited mobility of some people with RA, the 1:1 audio recorded semi-structured interviews were offered in the patient’s home (5/11), the outpatient department (2/11), the academic department (3/11) and over the telephone (1/11). Interviews lasted between 28 and 55 min. Subtle judgement between the researcher and supervisors led to recruitment termination when no new themes emerged from the interviews, illustrating that data saturation had been reached [19].

The focus group comprised of eight members (convenient sample) of the multidisciplinary team (2 doctors, 4 nurses and 2 podiatrists), led by GC and co-facilitated by third author HL, and lasted 45 min. All audio recordings (patients and focus group) were transcribed verbatim by staff from an externally contracted transcribing agency.

Interviews and focus group data were analysed separately using Framework analysis, a pragmatic applied research method [20]. The steps taken are outlined below.

1. Familiarisation, through immersion in the data: Transcripts, memo’s and audio –recordings were reviewed by the researcher to gain confidence about the content and subtlety of the gathered data [21].
2. Developing a thematic framework by identifying recurrent and important themes: Each transcript was read through in detail and codes, which captured items of data were developed and recorded using NVivo 10. Initial themes were developed from codes to facilitate iterative analysis recognising the interconnectedness of qualitative data [22].
3. Indexing and pilot charting: Using draft themes, transcripts and codes were re-examined and refined by combining and developing codes and sub themes into a draft framework. The refined framework accommodated all codes (278 codes from interviews, 96 from focus group).
4. Summarising the data in an analytical framework: Material was synthesised by refining descriptors and sub-themes to develop core themes and provide a succinct summary of the data. This process requires judgement about meaning, relevant importance of issues and considering and capturing implicit connections between ideas [23].
5. Synthesising data by mapping and interpreting: This was achieved by examining the relationship between core themes, associated literature and available theory.
6. Researcher reflexivity was maintained with the aid of diaries, memo’s and ongoing peer review. Robust peer discussion contributed to development of core themes and deviant cases [24] were studied in greater detail, ultimately leading to deeper and more inclusive findings. Independent validation of findings was sought at a local academic seminar. Respondent validation was agreed following presentation of the research findings during a patient group meeting (14.9.16) and a staff team meeting (22.11.2016), confirming that results resonated with participants experiences. Single counting was also employed to establish prevalence and representativeness of codes [24].

## Results

Participants’ socio-demographic characteristics are shown in Table 1 and details of the multi-disciplinary team in Table 2. No male patients were recruited onto the study, however RA does affect three times more women than men [25]. The rate of recruitment of those from Black African/Caribbean background reflects local demography, which has a high black (all origins) population [26]. As the rheumatology clinic does not have dedicated weight management specialist, it was not possible to capture her/his view in the focus group discussion.

**Table 1:** Patient characteristics

Patient Number	Age range	Ethnic Origin	BMI	Duration of RA
P1	50–60	Black British	32.98	2 years
P2	50–60	Black African	35.54	3 years
P3	40–50	White British	42.52	5 years
P4	70–80	White British	31.43	44 years
P5	60–70	Black Caribbean	40.11	10 years
P6	50–60	Black African	28.90	9 years
P7	60–70	Black African	28.96	36 years
P8	50–60	British Asian	33.19	6 years
P9	30–40	Black Caribbean	40.76	7 years
P10	60–70	White British	35.20	6 years
P11	50–60	White British	29.70	29 years

**Table 2:** Details of the multi-disciplinary team

Participant	Profession	Time in current post	Age range
01	Doctor	0–2 years	30–40 years
02	Nurse	0–2 years	40–50 years
03	Doctor	3–5 years	30–40 years
04	Nurse	0–2 years	40–50 years
05	Podiatrist	0–2 years	40–50 years
06	Podiatrist	0–2 years	20–30 years
07	Nurse	>5 years	40–50 years
08	Nurse	3–5 years	40–50 years

Three core themes emerged from the qualitative data analysis, including 11 sub-themes and 22 descriptors as shown in Table 3. Not all patients provided evidence for each sub-theme. Individual accounts are italicised with individual denominators (P for patient, number 1–11 i.e. P1) to illustrate that data was drawn from all study participants, and shown in Tables 4, 5, 6 and 7 under the relevant text. Commonly patients expressed contradictory views within their accounts and this will be apparent throughout the results sections, where number counting can exceed a total of eleven.

**Table 3:** Summary of Thematic Framework

Descriptor	Sub-theme	Core theme
Impact of weight gain Uncertainty of symptoms Effect on mood Internal struggle	Emotional response	The psychosocial impact of living with RA and obesity
Self-motivation Utilisation of public facilities	Active engagement with RA by patients	
Self-determination The role of food	Coping strategies	
Self-blame External barriers	Internal and external psychosocial challenges	
Unpredictability of the course of RA as a long term condition. Impact on lifestyle	Impact of living with RA	Challenges of living with RA and obesity
Frustration of weight Challenge of weight loss with RA	Problems of obesity alongside RA	
Engagement with Health Service. Working whilst living with RA.	Impact of external factors	
Operational expectations Psychosocial expectation	Expectations of a bespoke weight management programme	Considerations about future weight management programmes
Practical requirements of support for weight management programme. Views on technology in relation to a weight management programme.	Practicalities of designing and running a weight management programme	
The role of peer support Group dynamics	Purpose of weight management group	
	Awareness of obesity as a public health issue	

The data analysis of the focus group data also revealed three core themes with 6 sub-themes, which will be presented similarly to the interview data, and will include participants’ professional status.

### The psychosocial impact of living with RA and obesity

The core theme “the psychosocial impact of living with RA and obesity” was discussed by all interviewees. Patients talked about their emotional response to the impact of weight gain with RA, how they engaged with their RA to make them feel better physically and emotionally, coping strategies, which emerged in response to living with RA, and internal and external challenges, which caused emotional distress and its accompanied psychosocial impact.

All (11/11) patients described a negative emotional response to living with both conditions (Table 4, account 1). Uncertainty of symptoms caused emotional distress for 10/11 patients. When combined with the effort of lose weight, this commonly turned into a ‘negative’ mood experience for 9/11 patients, which could lead to an ‘internal struggle’, discussed by 5/11 interviewees. Many patients (7/11) verbalised how they motivate themselves and actively engage with their self-management (Table 4, account 2) and some (6/11) reported benefits from using public facilities, e.g. swimming to attempt to reduce the impact of symptoms and improve physical and emotional wellbeing (Table 4, account 3). Coping strategies to help manage living with the dual conditions were identified in many accounts (9/11) and food was recognised as a short term coping strategy in response to the stress of living with RA by the majority of participants 7/11 (Table 4, account 4). Self-blame (9/11) and the role of external barriers (6/11), were attributed to the multiple difficulties faced by those with RA, and a stigmatised condition such as obesity (Table 4, account 5).

**Table 4:** The Psychosocial impact of living with RA and obesity

*“There was a time, I really felt I’d given up now. I tried everything [to lose weight], I’ve done the gym part. You can’t lose it (weight) and things. You know, you’re limited because of what you’ve got [pain/fatigue/unpredictable symptoms] and your illness [RA]. You feel helpless”*. (P8)
*“Because sometimes I’m on the cross-trainer and I’m in so much pain I want to come off but I’m trying to say to myself “I mustn’t give up, I must keep going.”* (P5)
*“So I’m swimming now. I feel great now”.* (P9)
*“And in case of arthritis, sometimes it’s so frustrating you don’t even see yourself eating. Because you sit in one place, you can’t get up, you grab it and eat it [food]. You don’t realise you putting on the weight”.* (P6)
*“You might think other people are looking at you. You’re that size. But within yourself, you just keep quiet and suffer”.* (P8)

### Challenges of living with RA and obesity

Living with RA creates specific practical challenges not experienced by the general population when trying to lose weight. Characterised by often unpredictable symptomatology, life with RA presents a number of demands, such as difficulty planning for the future, or trying to balance symptoms and disease self-management (Table 5, account 1). Many (8/11) expressed concern about carrying extra weight and factors such as pain (Table 5, account 2) and potential side effects of medications (Table 5, account 3), which increased the challenge of addressing weight gain for the majority of patients (9/11).

External factors such as an ongoing relationship with the medical team and the hospital, and the ability to work are important considerations for participants. Many (7/11) reported that they had never had a conversation with doctor or nurses about their weight during outpatient clinic consultations. Patients tend to excuse clinicians, missed opportunities to address their obesity and recognised the limitations of a service where the patient and provider had to prioritise problems within the commonly limited consultation time (Table 5, account 4). 8/11 interviewees talked about the positive impact of continuing to work despite numerous physical challenges, e.g. distraction from uncomfortable symptoms of RA (Table 5, account 5).

**Table 5:** Challenges of living with RA and obesity

*“And you never know when it’s going to affect you. Aches and pains. You can feel great one day and some days you wake up and think “This is a good day”. The next day you think, “What did I do yesterday? I wasn’t actually that much more active, but certainly my ankle is hurting”.* (P11)
*“I love exercise but I’d be able to exercise and stuff, but I have to watch what I do because I get so tired. And so much pain, and the weight just will not come off”.* (P3)
*“Both of them go together because...if it’s not because of the medication, you are eating too much. But because of the medication, especially the one I’m taking, Methotrexate, at first I feel sick. Then I don’t even see myself eat. I really eat”.* (P6)
*“They’re [clinicians] on auto-pilot. They’re stressed! And not everyone is going to understand [their stress] like I understand. But they’re stressed, they’re overworked, they’re under pressure”.* (P9)
*“Also with rheumatoid, it’s the mind-set and sometimes your tiredness doesn’t make you want to get up and do things. I mean, I always do because my motivation is I like my work, I get out and do it. I want to get on. I’m self-employed, so I need to work. And that’s helped actually”.* (P11)

### Considerations about future weight management programmes

This final core theme emerged in response to questioning patients about the potential benefits and barriers of a bespoke weight management intervention for those with RA. All patients (11/11) spoke in detail about the operational and psychosocial expectations of an individual tailored weight management intervention (Table 6, account 1). The potential role for digital technology in providing access and education to patients who may struggle to physically attend a programme was articulated and many interviewees (8/11) stated the important role of new technology (Table 6, account 2) as part of the intervention. The majority (8/11) talked positively about group sessions and expressed clear benefits of providing peer support during the weight management programme (Table 6, account 3). A minority (3/11) however preferred to independently address their weight and favoured no involvement with fellow patients (Table 6, account 4). All (11/11) reported that group sessions would be helpful to some people and stressed the potential benefit for patients who are harder to reach. The majority (7/11) recognized a role for public health staff in tackling obesity (Table 6, account 5).

**Table 6:** Considerations for future weight management programmes

*“No it [the programme] wouldn’t be just about the weight. It would be about how they’re feeling, how their bodies feel, how they’re [the patients] coping and stuff like that”.* (P5)
*“Why not? It is a world of IT, so why not? I mean people are capable of using such things, if they’ve got the means. Why not?”* (P8)
*“I think that meeting other patients with RA with similar problems is a really great motivator. That’s what I find, you know, helps me. It’s when you have other people to motivate you along and who also know the struggles of the condition”.* (P3)
*“Coming back to that thing [trying to lose weight with RA] of you know, you’re not feeling great, and you just think “God, if you had to get up and exercise.” and someone says, “You’re in this group, you’ve got to get there every day.” It would probably do my head in”.* (P11)
*“I think this is a good thing [developing a weight management intervention for those with RA]. But it’s bigger than that. I think it requires everyone to put their hands in, not just XX Hospital or the NHS. It requires us to put this to the government and say, “Well, what are you going to do [about obesity as a public health issue]?”* (P9)

### Results from focus group with multidisciplinary rheumatology team members

The Focus group data generated three core themes: i) the relationship between provider and patient, ii) the relationship between the provider and the host institution in relation to the development of a future weight management programme, and iii) the social and political context of obesity.

Professional uncertainty in broaching the subject of weight gain was expressed by 5/8 providers who reported to be at times, ill prepared to address obesity sensitively and effectively with patients (Table 7, account 1). Staff described different responses to patient behaviour, which ranged from negative (2/8) and impatient (3/8), to more compassionate (3/8) (Table 7, account 2).

**Table 7:** Focus group quotations

*“You know, you start talking about it [obesity] and you feel it’s a very personal issue, and I don’t know, I just start to change the subject because you want them [patients] to be engaged in the process [weight loss] you don’t want them [the patients] to be put off. It’s difficult”.* (P9 Doctor)
*“I watch them drink the tea and put in a lot of sugar. I said that “If you know you’re overweight so you should reduce [sugar intake].” But they say, “I can’t. I can’t.” It’s a habit. They know they should do but it’s hard to change”.* (P5 Podiatrist)
*“And then, you know, you try to…send them [patients] to specialist physio, because they think this is the best exercise they could get and then of course they have to wait twelve months for an appointment, which...even if they have started to be engaged then you know, they can’t be engaged anymore because they have to wait a year. Because they can’t be seen”.* (P7 Nurse)
*“I’m not going to sit down and interact I don’t have the time to talk about these things [weight loss] with the patients. But I think they [patient] should address those things themselves because it’s their responsibility at the end of the day”.* (P6 Nurse)
*“But at the end of the day you have a massive public health problem with weight and you have to do something about it.*” (P9 Doctor)

Gaps in service provision about access to appropriate expertise, which made real, timely patient assistance difficult (Table 7, account 3), was highlighted, by 7/8 clinicians. Working within institutional limitations, such as lack of time and access to weight management expertise, provoked discussion and some conflict arose between participants about their role in this process. The question of who would be ultimately responsible, the patient, health care professional or the institution, to address weight management, provoked strong feelings amongst focus group members (7/8). Furthermore, this uncertainty added to the complexity and frustration experienced when trying to respond to patients concerns about with their weight gain. For example, half of the providers (4/8) recommended for patients to identify commercial weight management programmes (Table 7, account 4) and half (4/8) suggested to explore the options with patients during the outpatient clinic consultation.

Although contributory factors beyond institutional level did not feature largely during the focus group discussion, it was recognised by the clinicians that patients, staff and the host institution operated within a political and social context that influenced public attitudes to weight gain and obesity, (Table 7, account 5).

## Discussion

As far as we are aware, this is the first attempt to examine the perspectives of patients and staff on the impact of living with RA and obesity and the subsequent potential barriers to weight management, from patient’s and provider’s perspectives through a qualitative study. The data generated a deeper understanding about specific problems experienced by those living with RA and trying to, address weight gain, i.e. pain and fatigue, and health care professionals working with RA patients i.e. lack of confidence to talk about weight issues.

Three key findings were identified from the six core themes, which may be helpful if a bespoke future weight management programme is to be developed for overweight/obese patients diagnosed with RA.

### Living with RA and obesity results in compounded emotional distress for patients

Findings in the literature highlighted how patients with RA experience significant emotional distress with a profound psychosocial impact [27]. This was confirmed in this study where an unpredictable condition can make planning of daily activities difficult. The considerable added work and practical challenges of managing obesity further compounded the emotional distress associated with RA. In addition, society, already inclined to stigmatise obesity [17] could have its negative presumptions i.e. laziness, validated, which can add to patients’ frustration and distress, where inactivity can be misunderstood as ambivalence or lack of commitment rather than symptom led.

### Rheumatology providers appeared inadequately equipped to address the weight management needs of obese RA patients

Another key finding from the focus group showed how the specialist multi-disciplinary team members appeared to be ill-equipped to address weight management in overweight or obese patients with RA, despite recognising its importance in their management and care.

It is notable that weight management is not something currently on the rheumatology clinician agenda, for example weight is not discussed in any recommendations for annual review. The paradox in the outpatient clinic where the study took place was that all patients were routinely weighed before every clinic visit. The failure of clinicians to comment upon weight highlights a culture where clinical practice has become constrained within pathways and protocols, whereby healthcare professionals feel uncomfortable straying from their defined remit. This collusion of speciality practices and individual reservations to approach a sensitive issue may on the surface appear as ambivalence, although on more considered reflection represent a far more complex health challenge.

### Concerns about the delivery of weight management programmes differ between patients and providers

Interview dyads where patients and providers are interviewed about the same topic, can highlight complimentary as well as contradictory perspectives [28]. Through asking the same questions of each group it was anticipated there would be some overlap in findings. This did not emerge. Concerns raised by the two groups were different. This study, similar to Befort’s (2006), illustrated how patients readiness to have weight management addressed, was higher than anticipated by clinicians [29].

All patients declared they would welcome professional and psychosocial support for overcoming their weight gain and were largely positive about a bespoke programme. However, they recognised at the same time the specific difficulties patients face to lose weight with a diagnosis of RA. Potential barriers to participation in weight management programmes tended to focus on external factors such as work commitments rather than lack of motivation. Interestingly, a minority preferred to maintain independence and self-manage their RA and weight gain without health professional input.

### Limitations of the study

This study provides detailed insight into experiences of living with RA and obesity and incorporates the patient and provider perspective. The research was carried out in one tertiary outpatient clinic located within an inner-city ethnically diverse population. As common in qualitative research, findings are not generalizable [30] although the themes identified can inform future studies.

No men were interviewed and, in fact, only one man was screened in the recruitment process (he declined to take part due to work commitments). This may not be surprising given that RA affects 1% of the population [31] and the small study sample. Nonetheless the male view would be essential in future research. Our sample is not representative of the UK (86% Caucasian [32]), and the diversity of our participants is related not solely to ethnic origin, but also to mixed social circumstances. Nonetheless, much of the patient’s experiences of living with a dual diagnosis shared similarities. Whilst there were some differences, particularly around rituals and expectations about food and eating, it is not possible to attribute them to ethnic origin. The diversity of patients interviewed is representative of the local population and highlights how crucial it is to understand this demographic context before developing interventions.

### Relevance for clinical practice

One of the key barriers identified by professionals was the lack of confidence to introduce, to patients, the topic of weight gain. Findings from this study suggest that until addressing obesity becomes routinely and systematically integrated into professional’s outpatient consultation agenda, it will remain largely unexplored and marginalised. Placing obesity centre stage in clinic visits, assisted by incorporating weight management expertise, and adequate resources may help address what up until now remains largely a taboo subject. After all, weight measurements are taken routinely by outpatient staff prior to each outpatient clinic consultation. Furthermore efforts to reduce the stigma and blame culture (discrimination) associated with obesity [29] need to be tackled not just at the clinic level but possibly at an institutional level. This would help address public health concerns and destigmatise this emotive topic for patients and providers alike, so it can be more freely discussed by all.

## Conclusion

Weight gain in the local RA outpatient clinic is on the increase, and has significant negative implications for patient physical and mental health. Findings from the focus group show that talking about weight management is commonly avoided due to time constraints, fear of upsetting patients and lack of knowledge about local available weight reduction programmes, all of which may contribute to a potentially outwardly ambivalent attitude. Where the general population may find the topic of weight gain embarrassing or uncomfortable to discuss [17] this does not appear to be the case with RA patients in our study, who seemed very aware of the negative impact on their health and clinical outcomes.

Patients unanimously welcomed the opportunity to discuss their weight problems and agreed that support from their health care providers, would be welcome. A bespoke weight management, programme, which recognises and incorporates into its design, the added psychosocial impact and practical challenges associated with living with the dual diagnoses, was seen as an efficient way of addressing obesity, with supportive staff and peers.

## Abbreviations

BMI: Body Mass Index
RA: Rheumatoid arthritis

## References

[1] Public Health England. UK and Ireland prevalence and trends. 2014. http://webarchive.nationalarchives.gov.uk/20170110171021/https://www.noo.org.uk/NOO_about_obesity/adult_obesity/UK_prevalence_and_trends. Accessed 11 Jan 2017.

[2] Health Innovation Network (HIN). Tackling Obesity in South London. 2014. http://healthinnovationnetwork.com/system/resources/resources/000/000/019/original/Obesity_Discussion_Document_FINAL-_March_2014.pdf. Accessed 12 Dec 2015.

[3] Gremese E, Tolusso B, Gigante MR, Ferraccioli G (2014). Obesity as a risk and severity factor in rheumatic diseases (autoimmune chronic inflammatory diseases). Front Immunol.

[4] Daien CI, Sellam J (2015). Obesity and inflammatory arthritis: impact on occurrence, disease characteristics and therapeutic response. RMD open.

[5] Stavropoulos-Kalinoglou A, Metsios GS, Koutedakis Y, Nevill AM, Douglas KM, Jamurtas A (2007). Redefining overweight and obesity in rheumatoid arthritis patients. Ann Rheum Dis.

[6] Sandberg ME, Bengtsson C, Kallberg H, Wesley A, Klareskog L, Alfredsson L (2014). Overweight decreases the chance of achieving good response and low disease activity in early rheumatoid arthritis. Ann Rheum Dis.

[7] Uutela T, Kautiainen H, Jarvenpaa S, Salomaa S, Hakala M, Hakkinen A (2014). Waist circumference based abdominal obesity may be helpful as a marker for unmet needs in patients with RA. Scand J Rheumatol.

[8] Arranz LI, Rafecas M, Alegre C (2014). Effects of obesity on function and quality of life in chronic pain conditions. Curr Rheumatol Rep.

[9] Edwards RR, Calahan C, Mensing G, Smith M, Haythornthwaite JA (2011). Pain, catastrophizing, and depression in the rheumatic diseases. Nat Rev Rheumatol.

[10] Nikiphorou E, Norton S, Carpenter L, Dixey J, Walsh DA, Kiely P (2016). Secular changes in clinical features at presentation of rheumatoid arthritis: increase in comorbidity but improved inflammatory states. Arthritis Care Res.

[11] Colligan G (2017). Obesity and its impact on rheumatoid arthritis: a public health concern that cannot be ignored. Rheumatology.

[12] Anandacoomarasamy A, Fransen M, March L (2009). Obesity and the musculoskeletal system. Curr Opin Rheumatol.

[13] Patton MQ (2002). Qualitative research and evaluation methods.

[14] Green J (2014). Qualitative methods for health research.

[15] Lempp H, Kingsley G (2007). Qualitative assessments. Best Pract Res Clin Rheumatol.

[16] Marshall MN (1996). Sampling for qualitative research. Fam Pract.

[17] Owen-Smith A, Donovan J, Coast J (2014). “Vicious circles”: the development of morbid obesity. Qual Health Res.

[18] Britten N (1995). Qualitative interviews in medical research. BMJ (Clinical research ed).

[19] Parahoo K (1997). Nursing research. Principles, process and issues.

[20] Ward DJ, Furber C, Tierney S, Swallow V (2013). Using framework analysis in nursing research: a worked example. J Adv Nurs.

[21] Furber C (2010). Framework analysis: a method for analysing qualitative data. Afr J Midwifery Womens Health.

[22] Ritchie J, Lewis J (2003). Qualitative research practice: a guide for social science students and researchers.

[23] Srivastava A, Thomson SB (2009). Framework analysis: a qualitative methodology for applied policy research. JOAAG.

[24] Seale C (1999). Quality in qualitative research. Qual Inq.

[25] Rheumatoid Arthritis: the management of rheumatoid arthritis in adults. 2009. https://www.nice.org.uk/guidance/cg79/chapter/Context. Accessed 11 Jan 2017.

[26] Office for National Statistics (ONS). 2012. https://www.ons.gov.uk/peoplepopulationandcommunity/culturalidentity/ethnicity/datasets/2011censussmallpopulationtablesforenglandandwales. Accessed 18 May 2016.

[27] Daker-White G, Donovan J, Campbell R (2014). Redefined by illness: meta-ethnography of qualitative studies on the experience of rheumatoid arthritis. Disabil Rehabil.

[28] Kendall M, Murray SA, Carduff E, Worth A, Harris F, Lloyd A (2009). Use of multiperspective qualitative interviews to understand patients’ and carers’ beliefs, experiences, and needs. BMJ (Clinical research ed).

[29] Befort CA, Allen Greiner K, Hall S, Pulvers KM, Nollen NL, Charbonneau A (2006). Weight-related perceptions among patients and physicians: how well do physicians judge patients’ motivation to lose weight?. J Gen Intern Med.

[30] Malterud K (2001). Qualitative research: standards, challenges, and guidelines. Lancet (London, England).

[31] Humphreys JH, Verstappen SMM, Hyrich KL, et al. The incidence of rheumatoid arthritis in the UK: comparisons using the 2010 ACR/EULAR classification criteria and the 1987 ACR classification criteria. Results from the Norfolk Arthritis Register. Annals of the Rheumatic Diseases. 2012. doi:10.1136/annrheumdis-2012-201960. 10.1136/annrheumdis-2012-201960 PMC3711368 22945499

[32] Office for National Statistics. Ethnicity and National Identity in England and Wales. 2011. https://www.ons.gov.uk/peoplepopulationandcommunity/culturalidentity/ethnicity/articles/ethnicityandnationalidentityinenglandandwales/2012-12-11. Accessed 17 Dec 2016.

